# Prediction of Poor Outcome in Patients with Acute Liver Failure—Systematic Review of Prediction Models

**DOI:** 10.1371/journal.pone.0050952

**Published:** 2012-12-14

**Authors:** Kama A. Wlodzimirow, Saeid Eslami, Robert A. F. M. Chamuleau, Martin Nieuwoudt, Ameen Abu-Hanna

**Affiliations:** 1 Department of Medical Informatics, Academic Medical Center, University of Amsterdam, Amsterdam, The Netherlands; 2 Pharmaceutical Research Center, School of Pharmacy, Mashhad University of Medical Sciences, Mashhad, Iran; 3 Tytgat Institute for Liver and Intestinal Research, Academic Medical Center, University of Amsterdam, Amsterdam, The Netherlands; 4 South African DST/NRF Centre of Excellence in Epidemiological Modelling and Analysis, c/o StIAS, Stellenbosch, South Africa; Albert Einstein Institute for Research and Education, Brazil

## Abstract

**Introduction:**

Acute liver failure is a rare disease with high mortality and liver transplantation is the only life saving therapy. Accurate prognosis of ALF is crucial for proper intervention.

**Aim:**

To identify and characterize newly developed prognostic models of mortality for ALF patients, assess study quality, identify important variables and provide recommendations for the development of improved models in the future.

**Methods:**

The online databases MEDLINE® (1950–2012) and EMBASE® (1980–2012) were searched for English-language articles that reported original data from clinical trials or observational studies on prognostic models in ALF patients. Studies were included if they developed a new model or modified existing prognostic models. The studies were evaluated based on an existing framework for scoring the methodological and reporting quality of prognostic models.

**Results:**

Twenty studies were included, of which 18 reported on newly developed models, 1 on modification of the Kings College Criteria (KCC) and 1 on the Model for End-Stage Liver Disease (MELD). Ten studies compared the newly developed models to previously existing models (e.g. KCC); they all reported that the new models were superior. In the 12-point methodological quality score, only one study scored full points. On the 38-point reporting score, no study scored full points. There was a general lack of reporting on missing values. In addition, none of the studies used performance measures for calibration and accuracy (e.g. Hosmer-Lemeshow statistics, Brier score), and only 5 studies used the AUC as a measure of discrimination.

**Conclusions:**

There are many studies on prognostic models for ALF but they show methodological and reporting limitations. Future studies could be improved by better reporting and handling of missing data, the inclusion of model calibration aspects, use of absolute risk measures, explicit considerations for variable selection, the use of a more extensive set of reference models and more thorough validation.

## Introduction

Acute liver failure (ALF), also known as fulminant hepatic failure (FHF), is a rare disease associated with a very high mortality ranging from 60 to 90% depending on the etiology and the clinical experience of the reference center [1]. An early and exact assessment of the severity of ALF together with a prediction of its further development is critical in order to determine the further management of the patient. Spontaneous recovery occurs in a minority of patients. Although liver support devices can be considered as a temporary treatment, in most cases liver transplantation (LT) remains the only life saving treatment of irreversible ALF [2]. LT has been shown to improve outcome, achieving survival rates up to 80% [3]. The timely prediction of spontaneous recovery helps prevent LT and also the need for lifelong immunosuppressive therapy. Timely assessing the likelihood of mortality is important for decisions on emergency liver transplantation. Due to severe shortage of liver donors it is of utmost importance to distinguish patients requiring transplantation from those who will survive by receiving only intensive medical care. Predicting whether the patient with ALF will require transplantation or will recover with medical management alone is difficult.

A number of prognostic models have been used for outcome prediction in ALF patients to select patients in need of LT. The most widely applied ones are the King's College criteria (KCC), Clichy criteria, and the Model for End-Stage Liver Disease (MELD), which was originally developed to estimate post-procedural mortality in cirrhotic patients undergoing transjugular intrahepatic porto-systemic shunts (TIPS) [1], [4], [5]. The models have shown inconsistent reproducibility, prognostic accuracy and therefore cannot be taken to reliably predict mortality in ALF and the need for a better prognostic model remains [6], [7], [1]. Other prognostic models originally developed to measure the severity of illness for patients admitted to intensive care units, like the Acute Physiology and Chronic Health Evaluation II (APACHE II), Sequential Organ Failure Assessment (SOFA) and Simplified Acute Physiology Score III (SAPS III) also have been applied in ALF patients. Cholongitas *et al*. [8] showed that scores used to quantify severity of illness such as APACHE II or to monitor organ dysfunction like SOFA can be also used as early prognostic markers in ALF patients. A recent comparison among KCC, MELD, SOFA and APACHE II scores in patients with acetaminophen-induced acute liver failure concluded that KCC had the highest specificity (0.83) but lowest sensitivity (0.47) and SOFA had the best discriminative ability (Area Under the Receiver Operating Characteristic curve, AUC = 0.79) [9]. McPhail *et al*. in a recent meta-analysis [10] considered aspects of methodological quality of studies reporting the performance of only the KCC model and restricted the analysis to only acetaminophen-induced ALF. Other validation studies of the most widely applied models (such as KCC, Clichy criteria, MELD) can be found in the literature [1], [4], [11], [12]. However, to date there is no systematic review on the newly developed prognostic models for ALF patients.

The objective of this review was to identify and characterize prognostic models developed to predict mortality of ALF patients, and assess the quality of their respective studies. In addition, we identify the variables used for development of the models. Our review provides recommendations for future research on prediction models for ALF patients.

## Methods

### Search strategy and data sources

We re-used the search strategy employed in our prior published systematic review on ALF definitions [13]. Briefly, Ovid Embase(R) (1980 to 2012), Ovid MEDLINE(R) (1950 to 2012) and Ovid MEDLINE(R) In-Process & Other Non-Indexed Citations (1950 to 2012) for journal articles were searched based on keywords in title, abstract and MeSH terms. The following query was used: (prognosis OR prognostic OR predict*) AND (acute liver failure OR fulminant hepatic failure OR acute liver injury OR acute hepatic failure OR (acute on chronic AND liver failure)). “Liver failure” and “prognosis” were used as MeSh terms. The final search considered studies published up to 01 January 2012.

### Inclusion and exclusion criteria

We included articles only when they reported original data from a clinical trial or observational study on patients with ALF and if one of their main objectives was either developing one or more new prognostic models or modifying existing ones for predicting outcome (mortality/survival or LT) for ALF patients. Validation studies (defined as studies that validated the performance of earlier published models, without modification, on new data sets) assessing the performance of the established prediction models KCC, MELD, and Clichy criteria were excluded.

All duplicate articles resulting from the query above were removed and only English articles were considered. In the first step we excluded conference abstracts, paper reviews, comments and case studies. In the next step irrelevant studies were excluded based on titles and abstracts, followed by exclusion of the remaining studies based on their full text.

Two reviewers independently screened the titles and abstracts. Discrepancies between the 2 reviewers were resolved by consensus involving a 3rd reviewer. Figure S1 displays the search flowchart.

### Data collection and analysis

The studies that were included were classified as either developing a new model or modifying an existing model. A study developing a new model was termed a *development study*, in that it described and assessed the performance of a prediction model that had not previously been published. Development studies had to include at least one newly developed model, but may also have included other established existing models, such as the KCC, MELD, Clichy criteria, or any other more recently developed model for comparison. A study was classified as *modifying* an existing model if it described and assessed a modified version of a previously published model by, for example, adding or removing variables.

For each of the included studies, the general study characteristics (e.g. setting, study year, inclusion criteria, outcome and patients' characteristics) and model characteristics (e.g. the reported intended use of the prognostic model, technique(s) used for development and performance and validation of the model) were extracted. The quality of the study was reported using a structured data collection form as proposed by Medlock *et al*. [14]. As before, two reviewers extracted and summarized data and scored the methodological and reporting quality. Discrepancies between the reviewers were resolved by involving a 3rd reviewer.

### Prediction models

We distinguished two types of prediction models: regression models (e.g. survival Cox model, linear or logistic model); or decision models (e.g. a decision tree, discriminant analysis, or a decision rule based on a score variable). For the description of the models we recorded: the timing of variable measurement (e.g. at admission or peak value during some interval), type of prediction model, strategy of model development and the final model itself in the form of a prognostic formula or a decision rule.

### Variables

All input variables (potential predictors or confounders that were considered during model development), also known as covariates, were listed for each prediction model. We also report on the final input variables that remained in the final model after employing a variable elimination strategy.

### Prognostic performance measures

The performance measures were divided into the following 4 categories:

1. Statistical measures of performance based on a given cut-off point that result in a binary prediction (0 or 1): sensitivity, specificity, positive predictive value (PPV), negative predictive value (NPV), predictive accuracy (PA), positive likelihood ratio (PLR) and negative likelihood ratio (NLR).
2. Discrimination measures assessing the ability of the model to assign higher predicted probabilities of the outcome (e.g. death or LT) in patients actually having the outcome than those not having it. The most common measure is the AUC (Area Under the Receiver Operating Characteristic (ROC) curve).
3. Calibration measures assessing the proximity between the predicted probabilities and the actual risk of a group of similar patients. For example, the model was considered to be well calibrated when 25% of patients with a predicted mortality of 25% did indeed have the event. Common measures of (mis)calibration are the Hosmer-Lemeshow statistics.
4. Accuracy measures assessing the average proximity of a predicted probability for an individual patient to his/her actual outcome. These include elements of both discrimination and calibration [15] and are typically measured by the Brier score or Brier skill score (which is the proportion of explained variance).

We required that a study report on at least 2 of the above-mentioned performance categories in order to score positively in the quality assessment framework on model performance.

Validation pertains to assessing the statistical performance of a model. In order for prognostic models to be used in clinical practice they need to be credible, and validation is an important component for reinforcing model credibility. Apparent validation (estimating performance on the same sample from which the model was developed) may be biased due to leveraging on coincidental correlations in the development sample. For studies developing new models it is imperative to at least internally validate them. Internal validation means estimating the performance that would be obtained from a different sample but from the same population of the developmental dataset. This can be obtained by either estimating performance on a separate reserved subsample from the developmental sample or by resampling techniques from the developmental set using bootstrap or cross-validation techniques. Validation performed on a separate prospective dataset from the same setting is called temporal validation, and validation on a dataset in a different setting is called external validation [16].

### Quality assessment

The framework proposed by Medlock *et al*. [14] which was in part based on Minne *et al*. [17] and Hayden *et al*. [18] was used for assessment of the quality of content of the studies and the assessment of the performance of the models.

The framework has two sections: a reporting quality section for assessing the quality of reporting in the study, and a methodological section consisting of a number of questions assessing the rigor of model development and validation.

The reporting score consisted of 19 items (38 possible points) including description of study population, choice of predictors/variables to test, missing data, outcome measure and model description including its intended use and performance measures.

The methodological score consisted of 6 items (12 possible points) including a sufficient number of cases to support the number of variables, a representative population and validation.

Each item in the framework was rated as no (N, with 0 points), partly (P, with 1 point), or yes (Y, with 2 points). For example, the setting and study period should be reported to score 2 points and when only one is reported it scores 1 point.

The higher the score in each part of the framework was considered as higher quality of the study in that part.

## Results

### Study characteristics

Searching the online databases resulted in 1233 articles. Initial screening of titles and abstracts resulted in 125 articles for full text review, of which 20 articles met our inclusion criteria and were included in this review.

Detailed characteristics of these studies are presented in Table S1. The smallest and largest study included 23 and 588 patients, respectively. Mean (SD) and median [IQR] of sample size was 159 (164) and 99 [60–171], respectively.

In one study, gender was not reported (17% of patients), and 59% of patients were female among the remaining studies.

Five studies were conducted prospectively, 6 studies retrospectively and in 9 studies the data collection method was not reported.

### Model characteristics

#### Models

The included studies reported on 46 models. Eighteen studies proposed 26 new models and were considered as development studies. In one study the modification of an existing model, the KCC and in one other study the MELD were proposed. These models are described in Table S1. Models were developed to predict mortality (11 studies), survival (3 studies), need for transplantation (2 studies), and the combined outcome of death or transplantation (in 4 studies).

The majority of the studies used logistic regression analysis (14/20), 3 used Cox regression ([19], [20], [21]), 1 used both logistic and Cox regression [22], 2 used linear regression ([23], [24]), and 1 discriminant analysis [25]. Most of the newly developed models used variables measured at admission; some studies ([3], [26], [27]) used both the admission and peak values of variables during admission. One study used variables measured at the time of onset of grade 3–4 HE [28], 1 study [29] at the time of diagnosis (defined as the time when the patient fulfilled the diagnostic criteria of ALF, in Japan), 1 study [24] at days 1, 4, 8 and 15 following diagnosis, and 1 study [20] at the time of testing the serum sample for IgM anti-HBc.

Three studies performed temporal validation of the model's predictive performance using a second cohort of patients from the same hospital admitted after those in the development set. One study performed internal validation of the model using the resampling technique of leave-one-out where for each patient P a model is developed based on all other patients and tested on P [25]. The remaining studies did not perform any internal, temporal or external validation of the predictive performance of their models.

The most common performance measures reported in the studies were: sensitivity, specificity, PPV or NPV (16 studies). The thresholds in these performance measures depended on specific covariate patterns, instead of absolute probabilities. For example, in a model with three risk factors, 3 PPVs were calculated corresponding to patients having any one, or any two, or all risk factors. The next most commonly used performance measure was the AUC (5 studies). None of the studies used Hosmer-Lemeshow statistics, the Brier score or the Brier skill score.

#### Variables

Table S2 in the supplement lists 103 prognostic indicators (input variables) that were considered as potential predictors in the developed models. The table also includes the cut-off values for obtaining categorical indicators from continuous ones, which varied among studies; otherwise, the indicator was used as a continuous variable. Table 1 lists the indicators considered in more than ten studies in addition to the number of studies in which the indicator was categorized. The most commonly used indicator was bilirubin (16 times), which was selected in 8 final models. Twenty-three of 103 variables were use as categorical variables. The most commonly categorized variable was prothrombin time (PT, 10 times).

**Table 1:** Summary of the most often studied indicators (n = number of studies).

Indicator	Considered for developing the model (n)	Selected in the final model (n)*	Represented as categorical (n)
Bilirubin total	16	8	9
Age	15	8	8
HE	15	9	9
PT	14	9	10
Creatinine	13	5	5
Sex	13	1	-
ALT	11	1	2

* if a study reported more than 1 model, a variable selected in at least 1 model was counted one time.

#### Quality assessment framework

Table S3 in the supplement shows the scores of each study in terms of reporting and methodological quality. Reporting scores ranged from 17 to 30 points (median 24 out of a maximum of 38). Items scoring lower on reporting quality pertain to lack of reporting on missing values, on performance measures, and on reporting predicted probabilities or mortality percentages for covariate patterns for at least two specific groups of patients. Only 6 studies reported missing values, four of which reported on the way they were handled, and only one quantified the number of missing values. Other items scoring lower include giving the reason for the choice of the initial variables in the model and reporting on the spread of the primary outcome measure (confidence intervals). The best scoring items of the reporting score include reporting on patients' characteristic, defining and including important variables, and reporting on the intended use and a type of the model.

Methodological scores ranged from 7 to 12 points (median 10) out of a maximum of 12. Validation was performed in only 4 studies (3 studies used temporal validation and 1 used internal validation relying on the leave-one-out method). The best scoring items of the methodological part of the framework was reporting on the number of patients, number of events and number of variables.

For example, for the first item of the methodological score study by Hadem *et al*. [23] a score of 2 points ( = Y, Yes) was assigned since the number of events per variable in the final model was sufficient (>10). Study [30] received 1 point ( = P, Partially) since the number of events per variable was between 5 and 10 and study [19] received 0 points ( = N, No) because this ratio was below 5. The number of events is defined as the *minimum* of the number of ALF cases and non-cases. The study by Hadem *et al*. [23] scored on almost all items of the methodological score the maximum 2 points, except for the validation of the model, for which it received 0 points. Points scored for each item were summed up and the maximum attainable methodological score was 12 and for the reporting score 38.

In an additional sub-analysis (not shown, available from the corresponding author) we investigated the association between the quality scores and each of year of publication, number of article citations, and (current) impact factor of the journal where the study was published. There was a positive significant association between the year of publication and the reporting (but not the methodological) quality score. This was most pronounced after 2005 (mean reporting score till 2005 was 21.7 and after 2005 was 26.9). There were no relevant associations between journal impact factor or citations with the (methodological and reporting) quality scores.

#### Comparison to other models

Ten studies, next to developing at least one new model, simultaneously compared the new models to other existing historical models such as the KCC, MELD, SAPS III and SOFA, but never to another recently developed model. The most often reported reference model was KCC (10 studies), followed by MELD (5 studies), SAPS III and SOFA (each 1 study). Performance superiority was based simply on showing that one or more of the following measures was larger than those of the reference model: sensitivity, specificity, PPV, NPV, PA, PLR, NLR (9 studies; reference model was KCC in 9 studies, MELD in 5 studies, SOFA in 1 study); or on AUC (3 studies; reference model was KCC in 3 studies, MELD in 3 studies, SAPS III in 1 study). All those studies declared that their models were better than the established models. One study [29] showed that the reference model (KCC), when used as a covariate together with CTLV/SLV (ratio computed tomography-derived liver volume/standard liver volume) was not statistically significantly (p<0.05) associated with the outcome. This study suggested also that the model based on CTLV/SLV was not inferior to MELD and KCC in terms of sensitivity, specificity, PPV, NPV, PA as reported in the literature.

## Discussion

### Principal findings

In this review we identified, summarized and assessed the quality of available models in the literature for prediction of poor outcome in adult patients with ALF. There is a marked heterogeneity in the included studies and models (shown in Table S2 in the supplement) in terms of: variability of the characteristics of included populations, inclusion criteria, mortality rates, outcome (inclusion or exclusion of transplanted patients), considered predictors, and choice of reference models for comparison. Of note, 45% of the studies did not report whether data were collected prospectively or retrospectively. Although all studies aimed at including ALF patients, the definition of this disease differs among studies [13]. Despite this heterogeneity some general remarks can be made.

Model development usually relied on regression analysis, including logistic, linear or Cox regression (survival analysis). The models were usually constructed from clinical and/or demographic data to predict mortality or survival, only four studies used the combined outcome of death and transplantation. Generally the intended use of the developed models was clear, namely supporting decisions on whether to perform transplantation. However, half of the studies included small samples (<100 patients) and performed no internal, temporal or external validation. None of the studies reported on how well the model was calibrated. This is notable, as decision makers need to know how well a predicted probability corresponds to the true risk in the population. All studies comparing newly developed models with the “standard” models like MELD and/or KCC, SOFA and SAPS reported improved performance on these established models based on sensitivity, specificity, PPV, NPV, PA, PLR, and NLR. However, only 3 studies used additionally the AUC, and there are no calibration-related comparisons. Surprisingly the commonly used Clichy criteria were not used as a reference standard in the included studies. In general the apparent arbitrariness of selecting the reference models raises concerns about reporting bias. It will be useful when future studies attempt to compare the developed models to a standard set of other published reference models, including newly developed ones, for the same population.

### Strengths and limitations

To our knowledge this is the first systematic review exclusively dedicated to the assessment of the performance of newly developed prognostic models for adult patients with ALF. In our former review [13] we reported that there is a wide diversity in ALF definitions used in the literature, which hinders comparability and quantitative analysis among studies. In this review we identified and characterized newly developed prognostic models of mortality for ALF patients and assessed the quality of their respective studies. Our search has been extensive and we used an earlier published framework for quality assessment [14].

In the systematic review by Craig *et al*. [31] 14 studies were included which test the association of variables with poor outcome in paracetamol-induced ALF patients. The quality of these studies were assessed semi-quantitatively using a coarse grading system (poor, moderate, good or excellent) along six potential sources of bias in prognostic studies [18]. Another recent meta-analysis by McPhail *et al*. [10] assessed the quality of studies validating KCC and restricted to acetaminophen-induced ALF. These studies are excluded in our review. Our review included studies pertaining to ALF patients independently of aetiology; it extends the quality assessment framework in [12] and distinguishes between reporting and methodological quality; it includes only prognostic models, not merely tests of pre-selected variables; and it considers newly developed models rather than validation of existing ones.

We intentionally excluded external validation studies like [1] and [4] since these would correspond to the already established models (MELD, KCC, Clichy Criteria) and there are already existing reviews on those models such as [10], [11], [12], [32]. In our review we did include the studies on the development of KCC [27] and Clichy Criteria [20] for ALF patients. There is no journal paper describing the development of the MELD for ALF patients. MELD has been primary designed to estimate short-term post-procedural mortality of cirrhotic patients undergoing transjugular intrahepatic portosystemic shunt [33], and later in patients with end-stage chronic liver disease of diverse aetiology and severity [34]. In 2002 MELD was implemented in the USA by the United Network for Organ Sharing for organ allocation in patients with chronic liver disease awaiting LT [21]. In 2003 in a conference abstract Aydin *et al*. [35] suggested that MELD can be used as a complementary tool to predict prognosis in ALF patients. MELD received increasingly more attention and was applied as predictor for ALF patients [21]. We hence consider this latter paper as the first one officially dedicated to ALF patients and included in our review.

### Implications and recommendations

In order to be clinically useful, predictive models need to be credible. This credibility is largely dependent on the model validity. As reported by Cook [15] evaluation of models for medical use should take the purpose of the model into account. Evaluation of prognostic models should not be confined to only ROC curve analysis, but should assess various relevant performance measure covering at least both discrimination and calibration.

Validation of a model is necessary to provide evidence of its potential to accurately predict outcomes especially at the individual patient level. As reported by Altman *et al*. [36] un-validated models should not be used in clinical practice. However, our review revealed that the great majority of models have not even been internally validated nor has their calibration been assessed. Surprisingly, only four studies [37], [24], [27], [25] performed some form of validation of their models. Future studies should provide calibration performance assessment (using e.g. the Hosmer-Lemeshow goodness-of-fit statistics or, even better, the Brier score) and should undergo adequate internal validation, a good choice would be the use of bootstrap techniques to this end. Moreover, in an additional search we found than none of these models has been externally validated elsewhere.

Many studies (e.g. [29], [24]) have considered the transplanted patient group and non-survivors in one group. Patients who receive transplantation may consist of the most severe cases and would have died without transplantation. Those transplanted patients are probably similar to those who do not survive. A separate analysis should be performed to compare the transplantation patient characteristics to the non-survivor group, like in the study of Dabos *et al*. [38]. When the groups are similar one can consider forming the group with the combined outcome “transplanted or died,” at least for sensitivity analysis.

In the literature different kinds of models are applied, like logistic regression or Cox regression. One should consider the aim of the study when developing a model. Logistic regression is appropriate when the aim of the study is prediction of an event (mortality or survival) without regard to how long it took for the event to occur. When the aim of the study is to predict the time until event occurrence then the Cox regression is an appropriate choice.

Most of the studies did not report on missing data. Only five studies [3], [27], [37], [38], [39] reported how they handled the missing values. Excluding cases with missing values reduces the sample size and can bias the results. One should compare cases with missing and non-missing values on other known variables to check for bias. In addition (multiple) imputation of the missing variables should be considered [40].

There was marked heterogeneity in the included variables. The majority of the evaluated variables where used only once (65/99). The reason for choosing the initial variables was often not clear. Only four studies [3], [20], [28], [29] stated this reason. Selection of the variables entered into the multivariate analysis was mostly based on the significant results from univariate analysis. When the number of variables is not excessive one should consider whether variable selection is required, and if so, consider using an information criterion (such as the Akaike Information Criterion) in the (e.g. stepwise) selection process instead of relying on p-values ≤0.05 in univariate analysis.

Many studies did not report why and how the continuous variables were categorized. Categorization causes information loss and has consequently less precise coefficient estimation and reduced statistical power to detect an association between the variable and patient outcome. For example, in the study of Tylor *et al*. [19] when creatinine was considered as a continuous variable it was significantly associated with mortality in univariate analysis, but not when it was used as a categorical variable. Categorization of continuous variables without compelling reasons should hence be avoided. If categorization is unavoidable then the choice for the cut-off points should be motivated.

Because many variables indicate categories, or are represented as such, most models will have only a small set of different predicted probabilities. For example when using 2 binary variables in the model there are at most 4 discrete different predicted probabilities and no continuous range. Continuity in the predicted probability would allow models to exhibit smooth behaviour in which small changes in the covariate values are reflected by small changes in the outcome. This leads to better distinctions among patients.

In our systematic review we extracted variables which were used for constructing the models (Table S2). These variables comprise a huge number of possible predictors. Based on clinical experience and from a theoretical point of view we would suggest to include variables involved in the pathophysiology of ALF as well as age and grade of HE. Specifically the following variables may be considered: plasma ammonia, which rises as a consequence of impaired hepatic urea synthesis and contributes to the development of HE; plasma bilirubine, which increases as a consequence of impaired biliary excretion and coagulopathy as a consequence of decreased protein synthesis, especially of clotting factors, expressed by INR. We also think that some aspect of impaired capacity of the ALF patient to maintain metabolic homeostasis should be considered. In this respect plasma lactate might be relevant. In addition the biomarkers of the inflammatory response of ALF might be considered: e.g. plasma ratio of IL6/IL10.

Due to the methodological and reporting quality limitations generally encountered in the included studies we would recommend to develop new models that consider most important relevant variables and follow the methodological and reporting recommendations presented in this study. The predictive performance (and hopefully clinical value) of such models will need to be tested in prospective large cohorts of ALF patients with different etiologies.

In our sub-analysis we concluded that there was no relevant association between any of impact factor and citations with any of the quality of methodological and reporting score but that there was a clear positive association between year of publication and reporting score.

### Conclusion

This systematic review provides an overview of models for prediction of poor outcome in patients with acute liver failure. These prognostic models were developed to support clinicians' decisions, but they should be improved before being clinically useful. Future studies could be improved by paying more attention to (internal) validation, the inclusion of model calibration aspects, better consideration of the transplantation patient group, better reporting and handling of missing data, use of absolute risk measures, explicit considerations for considering and selecting predictors, the use of a more extensive set of reference models, and the inclusion of continuous variables without categorizing them, as well as clear reporting on the study design. It is hoped that the results of this review can be useful for developers of future prognostic models for ALF patients.

## Supporting Information

Figure S1 **Search flowchart.**
(TIF)

Table S1 **Detailed summary of the included studies.**
(DOC)

Table S2 **Summary of input variables used in the studies.**
(DOC)

Table S3 **Quality assessment scores of the included studies.**
(DOC)
