# Supplementary figures and images for: Prediction of Poor Outcome in Patients with Acute Liver Failure—Systematic Review of Prediction Models
Source: PLoS One. 2012 Dec 14;7(12):e50952. doi: 10.1371/journal.pone.0050952 (PMC3522683; doi:10.1371/journal.pone.0050952)

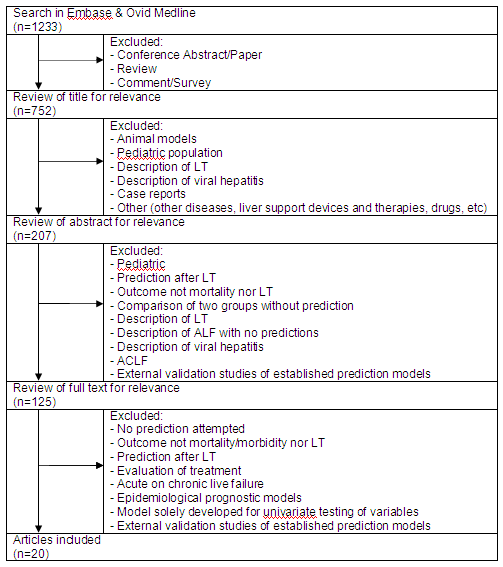

Supplement: Figure S1 — Search flowchart. (TIF) [file pone.0050952.s001.tif]
