# Supplementary material for: Prediction of Poor Outcome in Patients with Acute Liver Failure—Systematic Review of Prediction Models
Source: PLoS One. 2012 Dec 14;7(12):e50952. doi: 10.1371/journal.pone.0050952 (PMC3522683; doi:10.1371/journal.pone.0050952)
Supplement: Table S1 — Detailed summary of the included studies. (DOC) [file pone.0050952.s002.doc]

**Supplement Table S1**: Detailed summary of the included studies

| Study | setting | population | outcome, time of measurement, purpose | model and validation | performance  (comparison of models) | conclusion |
| --- | --- | --- | --- | --- | --- | --- |
| Acute liver failure in Scotland between 1992 and 2009; incidence, aetiology and outcome  Bretherick et al.  Q J Med 2011; 104:945–956 | Scottish Liver Transplant Unit (SLTU) in the Royal Infirmary of Edinburgh  November 1992 - March 2009  prospective | 514 patients  **age at inclusion**:  mean 39.7±14.8  **sex:**  220M/294F  **inclusion criteria**:  ALF patients, where ALF is defined as by O’Grady -explicit in this definition is the speed of onset of the condition and presence of HE | **outcome**:  survival  **mortality:**  275 (29%) died  **time of measurement**:  admission  **purpose**:  to describe incidence, aetiology and outcome data for Scotland since the inception of the Scottish Liver Transplant Unit (SLTU) in 1992. | **statistical analysis:**  backward stepwise logistic regression  **model:**  1) Referring hospital  regression coefficients:  HE: 1,279  WBC count (×10^9^/l): -0.017  PT: -0.008  Creatinine (µmol/l): -0.002  Bilirubin (µml/l): -0.004  2) SLTU  regression coefficients:  HE: 1.542  Hemoglobin: 0.016  WBC count: -0.032  Platelets: 0.003  PT: -0.028  Potasium: -0.857  Urea: 0.064  Creatinine: -0.003  Bilirubine: - 0.005  ALT: 0.000  **validation**: No | OR (95% CI) of survival if encephalopathic (ALF) on admission to referring hospital: 0.24 (0.17-0.33)  OR (95% CI) of survival if encephalopathic (ALF) on admission to the SLTU  0.14 (0.11 - 0.20) | On admission to the referring hospital HE, serum creatinine and bilirubin were found to be associated with outcome. On admission to the SLTU more variables were found to be associated with outcome; HE, haemoglobin concentration, WBC, platelet count, PT, serum potassium, urea,  creatinine, bilirubin and ALT concentrations. |
| Antituberculosis Therapy–Induced Acute Liver Failure:  Magnitude, Profile, Prognosis, and  Predictors of Outcome  Kumar et al.  HEPATOLOGY 2010; 51:1665-1674 | Department of Gastroenterology, All India Institute of  Medical Science, New Delhi  January 1986 - January 2009  prospective | 70 patients  **age at inclusion**:  mean 32.87 ± 15.8,  median 27 (13-80)  **sex:**  21M/49F  **inclusion criteria**:  ALT-ATT patients with ALF defined by occurrence of HE within 4 weeks of symptoms in absence of preexisting liver disease. ATT-ALF was diagnosed if the patient with ALF had a history of consumption of at least two of the three first-line hepatotoxic drugs (isoniazid, rifampicin, and pyrazinamide) for a minimum period of 1 week and if the patient’s sera tested negative for evidence of known hepatitis virus(es) infection (HAV, HBV, HCV, HEV), and absence of any other identifiable cause of acute liver injury | **outcome**:  mortality  **mortality:**  47 (67.1%) died  **time of measurement**:  admission  **purpose**:  to evaluate magnitude, course, complications, and prognostic model in patients with ATT-ALF in an endemic region of tuberculosis. | **statistical analysis:**  logistic regression with stepwise selection procedure  **model:**  1) * 3 factors independently predicted mortality:  - serum bilirubin (≥10.8 mg/dL)  - PT prolongation (≥26 seconds),  - grade III/IV HE  2) KCC  3) MELD  **validation**: No | AUROC (95% CI):  serum bilirubin: 0.737 (0.611-0.863)  PT: 0.717 (0.57-0.86)  OR:  serum bilirubin (≥10.8 mg/dL): 13.08,  PT (≥26 seconds): 6.81,  grade III/IV HE: 6.08  Sensitivity (%), Specificity (%), PPV (%), NPV (%), PA (%):  1) At least one of three*  100, 13.6, 66.7, 100, 67.7  At least two*  81.1, 72.7, 83.3, 69.6, 77.9  All three*  40.5, 100, 100, 50, 62.7  2) KCC  34.04, 74, 72.7, 35.4, -  3) MELD (cut-off 33):  73, 66.6, 86.6, 50, -  MELD calculated for 21 patients | Serum bilirubin, PT prolongation and grade of HE at presentation were independent predictors of mortality. The mortality rate was found to be increased with the increasing number of adverse prognostic factors.  The KCH criterion  was found unhelpful in ATT-ALF because it yielded a  low sensitivity. In a subgroup analysis, MELD score discriminated survivors from non-survivors  with a reasonable sensitivity and specificity. The presence of any two of three  poor prognostic factors yielded the best  prognostic power. |
| A new prognostic formula for adult acute liver failure using computer tomography-derived hepatic volumetric analysis  Yamagishi et al.  J Gastroenterol 2009; 44: 615–623 | Keio University Hospital, Japan  January 1999 - May 2007.  retrospective | 30 patients  **age at inclusion**:  45.7 (19–83) survivors  46.7 (22–69) death+LT  **sex:**  14M/16F  **inclusion criteria**:  adult patients with ALF diagnosed according to the diagnostic criteria of the Inuyama Symposium in Japan: prothrombin time (PT) (%) less than 40% and developed HE greater than grade II within 8 weeks after the onset of symptoms | **outcome**:  need for LT  **mortality**:  6 died,  11 underwent LT  **time of measurement**:  time of diagnosis  **purpose**:  to assess the value of liver volumetry and to generate a new prognostic formula. | **statistical analysis:**  logistic regression stepwise regression model  **model**:  1) Z = -2.3813 - [0.15234 9xTB (mg/dl)] +  [4.5734 x CTLV/SLV]  2) KCC  3) MELD  **validation**: No | Z<0: estimated poor prognostic case  AUROC: 0.87783  OR(95%CI):  TB (>17mg/dl): 4.734 (-0.616–2.577)  CTLV/SLV (<0.80): 22.09 (0.179–3.453)  Sensitivity (%), Specificity (%), PPV (%), NPV (%), PA (%)  1) 94.1, 76.9, 84.2, 90.9, 80.0  2) KCC:  OR(95%CI): 0.7300 (-1.535 –1.044)  3) MELD (cut-off 30): NS | The CTLV/SLV ratio is a very useful marker for predicting the prognosis of adult ALF. The prognostic formula including only the CTLV/SLV ratio and TB is simple and useful and awaits validation in a future larger-scale prospective study.  Multiple logistic regression analysis identified that the CTLV/SLV ratio was independent predictor and KCC not.  The formula is not inferior to MELD and KCC, |
| Prognostic Implications of Lactate, Bilirubin, and Etiology in German Patients With Acute Liver Failure.  Hadem et al.  Clinical Gastroenterology and Hepatology 2008; 6:339–345 | Hannover Medical School,  Germany  1996 – 2005  retrospective | 102 patients  **age at inclusion**:  38 (16-74)  **sex:**  30M/72F  **inclusion criteria**:  patients fulfilled the diagnostic criteria of ALF: HE, acute-onset increase of INR >1.5, and absence of signs of chronic liver disease in clinical and ultrasound examination | **outcome**:  death or need of LT  **mortality**:  18 (18%) died,  45 (44%) underwent LT (5 (5%) died)  79 survived 8 weeks after  admission at ICU (77%)    **time of measurement**:  admission  **purpose**:  to evaluate prognostic parameters in a central European cohort. | **statistical analysis:**  linear regression analysis  **model:**  1) BiLE score:  Bilirubin (µmol/L)/100  + lactate (mmol/L)  + 4 (in case of indeterminate ALF, Budd-Chiari syndrome, or phenprocoumon toxicity)  - 2 (in case of acetaminophen toxicity)  + 0 (in case of any other ALF etiology)  2) KCC  3) MELD  4) SAPS III  **validation**: No | AUROC ± SE (95%CI):  BiLE 0.87±0.04 (0.80-0.95)  MELD 0.71±0.05 (0.61-0.82)  SAPS-III 0.68±0.59 (0.57-0.79)  Sensitivity (%), Specificity (%), PPV (%), NPV (%):  1) BiLE (cut-off 6.9): 79, 84, 89, 71  2) KCC: 58, 82, 83, 55  3) MELD (cut-off 32):  65, 69, 77, 55 | The simple, combined BiLE score emerged as the best predictor of poor outcome in the patient cohort and should be prospectively evaluated in other populations.  AUC values revealed a better performance of the BiLE score in comparison with KCC, MELD score, and the SAPS III score. |
| Early Indicators of Prognosis in Fulminant Hepatic Failure: An Assessment of the Model for End-Stage Liver Disease (MELD) and Kings College Hospital Criteria.  Dhiman et al.  Liver Transplantation 2007; 13:814-821 | Emergency Medical  Ward  January 1996 - June 1998  retrospective | 144 patients  **age at inclusion**:  28.6±12.3 survivors  33.4±15.8 non-survivors  **sex:**  62M/82F  **inclusion criteria**:  onset of HE occurring within 12 weeks of onset of jaundice and was further subclassified into hyperacute (interval 0-7 days), acute (interval 8-28 days), and subacute (interval 29 days-12 weeks) liver failure | **outcome**:  mortality  **mortality**:  92 (63.9%) died  **time of measurement**:  admission  **purpose**:  to compare MELD and KCH criteria with other early clinical prognostic indicators (CPI). | **statistical analysis:**  logistic regression analysis with backward elimination procedure  **model:**  1) Presence of any 3 of 6 CPI  age ≥50 yr, JEI >7 days, grade 3 or 4 HE, presence of cerebral edema, prothrombin time ≥35 s, and creatinine ≥1.5 mg/dL  2) KCC  3) MELD  **validation**: No | c-statistic [95% CI]:  1) 3 CPI: 0.802 [0.726-0.878]  2) KCC 0.676 [0.588-0.764],  3) MELD 0.717 [0.636-0.789]  Sensitivity (%), Specificity (%), PPV (%), NPV (%), PA (%):  1) Any 1 factor:  100.0, 9.6, 66.2, 100.0, 67.4  Any 2 factor:  97.8, 42.3, 75.0, 91.7, 77.8  Any 3 factor:  73.9, 86.5, 90.7, 65.2, 78.5  Any 4 factor:  30.4, 100.0, 100.0, 44.8, 55.6  2)KCC: 76.1, 67.3, 80.5, 61.4, 72.9  3)MELD (≥ 33): 46.7, 88.5, 87.8, 48.4, 61.9 | MELD and KCH criteria are not as useful as a combination of other early CPI in predicting adverse outcome in patients with FHF due to acute viral hepatitis.  Presence of any 3 CPIs is  superior to MELD and KCH criteria in predicting the  outcome. |
| Utility of the MELD, KCC and a New In-Hospital Mortality Score in the Prognosis.  Pelaez-Luna et al.  Transplantation Proceedings 2006; 38, 927–929 | Department of Gastroenterology, Instituto Nacional  de Ciencias Médicas y Nutrición Salvador Zubirán, Mexico City,  Mexico.  1983 – 2004  retrospective | 58 patients  **age at inclusion**:  FHF: 37 ± 14  SFHF 37 ± 18  **sex:**  17M/41F  **inclusion criteria**:  ALF patients; FHF and SFHF patients, FHF: development of HE occurring within 2 weeks of the onset of jaundice; SFHF: development of HE 2 weeks to 3 months after the appearance of jaundice | **outcome**:  in-hospital mortality  **mortality**:  28 died  **time of measurement**:  during admission  **purpose**:  to evaluate and compare a new ALF in-hospital mortality prediction score versus KCC and MELD score | **statistical analysis:**  logistic regression (Wald step forward) model  **model:**  1) ALFIHM =  0.714 + 0.02 (TB) + 0.03 (APACHE II score) × 10  2) KCC  3) MELD  **validation**: No | OR(95%CI):  total bilirrubin: 1.2 (1.05 - 1.3 APACHE II: 1.3 (1.13- 1.5)  ALFIHMS cut-off point >15 points is associated with an in-hospital mortality probability >50%.  PPV (%), NPV (%):  1) ALFIHMS (>15): 80, 82  2) KCC: 59, 58  3) MELD (>25): 71, 73 | ALFIHMS has higher prognostic accuracy, is more specific and more sensitive and with higher PPV and NPV than MELD and KCC in ALF. |
| Prognostic implications of hyperlactatemia, multiple organ failure, and systemic inflammatory response syndrome in patients with  acetaminophen-induced acute liver failure  Schmidt and Larsen  Crit Care Med 2006;  Vol. 34, No. 2 | Department of Hepatology  at Rigshospitalet, Denmark  January 1999 - June 2004  retro/prospective NR | 101 patients  **age at inclusion**:  49 (12–75)  **sex:**  32M/69F  **inclusion criteria**:  severe acetaminophen-induced FHF defined as the development of HE grade 3–4 | **outcome**:  mortality  **mortality**:  48 died,  6 underwent LT (1 died, 5 survived)  **time of measurement**:  - admission  - at the time of onset of grade 3-4 HE  **purpose**:  to evaluate arterial lactate as a prognostic marker in acetaminophen-induced  fulminant hepatic failure and to analyze its relationship to known  causes of hyperlactatemia such as multiple organ failure and inflammation | **statistical analysis:**  logistic regression analysis  **model:**   1. Modified KCC (+lactate) 2. KCC 3. SOFA   **validation**: No | Sensitivity (%), Specificity (%), PPV (%), NPV (%), PLR (%), NLR (%):  1) Modified KCC:  - At the time of onset of grade 3-4 HE:  87, 44, 62, 77, 1.57, 0.29  - At any time:  91, 40, 61, 82, 1.52, 0.22  2) KCC  - At the time of onset of grade 3-4 HE:  53, 81, 75, 64, 2.94, 0.54  - At any time:  71, 77, 76, 72, 3.03, 0.38  3) SOFA score >12  - At the time of onset of grade 3-4 HE:  81, 68, 72, 78, 2.55, 0.28 | Applying the lactate modification of the KCH criteria did improve their sensitivity, but at the cost of a significantly reduced specificity.  The lactate modification of the KCC showed no obvious advantages over the existing selection criteria. |
| Fulminant Hepatitis A Virus Infection in the United States: Incidence, Prognosis, and Outcomes  Taylor et al.  HEPATOLOGY 2006; 44:1589-1597 | Acute Liver Failure Study Group (ALFSG) is a multi-center consortium of 24 sites  1/1/1998 - 9/15/2005  prospective | 29 patients  **age at inclusion**:  48±14  **sex:**  18M/14F  **inclusion criteria**:  the presence of coagulopathy (i.e., prothrombin time >15 seconds or international normalized ratio [INR] ≥1.5) and HE within 26 weeks of symptom onset in the absence of pre-existing liver disease | **outcome**:  death/transplantation  **mortality**:  4 died,  9 underwent LT  **time of measurement**:  admission  **purpose**:  - to identify the  presenting features associated with a poor prognosis (i.e., need for liver transplantion, or death) in HAV patients  prospectively enrolled in the ALFSG observational study.  - to develop an HAV specific prognostic  model from the ALFSG database and compare its performance to other published models including the King’s College criteria and laboratory MELD scores. | **statistical analysis:**  forward stepwise (Wald) Cox regression modeling  **model:**  1) *ALFSG index:  serum ALT <2,600 IU/L, creatinine >2.0 mg/dL, intubation, pressors  2) KCC  3) MELD  **validation**: No | AUROC:  1) ALFSG (4 factors): 0.538  ALFSG (≥3 factors): 0.766  ALFSG (≥2 factors): 0.899  ALFSG (≥1 factors): 0.781  2) KCC: 0.623  3) MELD (≥35): 0.707  Sensitivity (%), Specificity (%), PPV (%), NPV (%):  1)ALFSG index (4 factors):  8, 100, 100, 57  ≥ 3 Factors: 62, 94, 89, 75  ≥ 2 Factors 92, 88, 86, 93  ≥ 1 Factor 100, 56, 65, 100  serum creatinine (> 2.0 mg/dl):  54, 88, -,-  ALT (<2,600 IU/mL): 77, 75, -, -  2)KCC: 31, 94, 80, 62  3) MELD (≥35): 54, 88, 78, 70 | A prognostic index consisting of 4 clinical and laboratory features predicted the likelihood of transplant/death significantly better than other published models (KCC, MELD) suggesting that disease specific prognostic models may be of value in non-acetaminophen ALF. |
| A Biochemical Prognostic Model of Outcome in  Paracetamol-Induced Acute Liver Injury  Dabos et al.  Transplantation 2005; 80: 1712–1717 | Royal Infirmary of  Edinburgh, Edinburgh, Scotland, United Kingdom (Scottish Liver Transplant Unit)  1^st^ cohort:  January 1997 - December 1998  2^nd^ cohort:  January 1999 - December 2000  retro/prospective NR | 1^st^ cohort: 97 patients,  2^nd^ cohort: 85 patients  **age at inclusion**:  1^st^ cohort: 36.2±6.8  2^nd^ cohort: 35.7±6.4  **sex:**  1^st^ cohort: 50M/47F  2^nd^ cohort: 41M/44F  **inclusion criteria**:  paracetamol induced acute liver injury based on guidelines: a progressive coagulopathy where the prothrombin time in seconds was greater than the time after overdose in hours or a prothrombin time greater than 50 seconds at any time, or the presence of metabolic acidosis, hypoglycemia, renal failure or HE. The KCH criteria were applied continuously throughout the patients’ admission. | **outcome**:  death/ transplantation  **mortality**:  1^st^ cohort:  19 died,  6 underwent LT  2^nd^ cohort:  26 died,  9 underwent LT  **time of measurement**:  admission  **purpose**:  to develop a prognostic model of outcome for patients with paracetamol induced acute liver injury based on admission parameters | **statistical analysis:**  stepwise forward logistic regression  **model:**  1) biochemical criteria: (400×Pyruvate mmols/L) + (50×Phenylalanine mmols/L) –  (4× Hemoglobin g/dL)  2) KCC  **validation**: Yes | A value of<16 had an accuracy of 93% in predicting death correctly  Sensitivity (%), Specificity (%), PPV (%), NPV (%):  1) 91, 94, 91, 94  2) KCC (on admission): 41, 100, 100, 76  KCC (at any time): 88, 96, 94, 93 | Using admission characteristics the model is able to identify patients who die from paracetamol overdose FHF as accurately as KCC, but at a much earlier stage in their condition. |
| New Prognostic Scoring Model for Liver  Transplantation in Patients with Non-Acetaminophen-  Related Fulminant Hepatic Failure  Miyake et al.  Transplantation 2005; 80: 930–936 | Okayama University Hospital and 11 tertiary care centers with a liver transplantation program for fulminant hepatic  failure  1^st^ cohort:  January 1990 - March 2001  2^st^ cohort:  May 2001 - December  2003  retrospective study;  prospective validation | 1^st^ cohort: 80 patients,  2^nd^ cohort: 26 patients  **age at inclusion**:  1^st^ cohort: 45.5 (16-78)  2^nd^ cohort: 61.0 (19-81)  **sex:**  1^st^ cohort: 33M/47F  2^nd^ cohort: 10M/16F  **inclusion criteria**:  patients with FHF (criteria included the development of HE ≥ grade II within 8 weeks from the onset of initial symptoms, a prothrombin activity of less than 40%, and no previous chronic or alcoholic liver disease) | **outcome:**  2-week fatal outcome and need for transplantation  **mortality**:  cohort1:  48 (60.0%) died,  5 underwent LT  cohort2:  13 (50.0%) died,  4 (15.4%) underwent LT  **time of measurement:**  at the time of diagnosis (day 1), on days 4, 8, and 15  **purpose**:  predicting 2-week outcomes and determining the suitable timing for LT in patients with non-acetaminophen-related FHF | **statistical analysis:**  stepwise multiple linear regression  **model:**  1) day 1: 0.028 + 0.205 cause (HBV or indeterminate) + 0.301 SIRS (yes) + 0.342 ratio of T/D bilirubine (>2.0)  2) day 4: -0.158 + 0.440 HE (grade III or IV) + 0.203 SIRS (yes) + 0.269 total bilirubin (>15mg/dl) + 0.386 ratio of T/D bilirubine (>2.0)  3) day 8: -0.009 + 0.470 HE (grade III or IV) + 0.241 SIRS (yes) + 0.268 ratio of T/D bilirubine (>2.0)  4) day 15: -0.047 + 0.524 HE (grade III or IV) + 0.388 platelet count (≤10×103/mm^3^)  **validation:** Yes | The score 3 or more predicted prognosis.  The 2-week survival rate in patients scoring <2 was more than 80% in contrast to less than 30% in patients scoring ≥3.  11 of 24 patients scoring  <3 on day 1 had a poor 2-week prognosis. However, 9 of the 11 patients scored 3 or more on day 4. The score on day 4 may be more important in order to determine the suitable timing for LT  Sensitivity (%), Specificity (%), PPV (%), NPV (%):  87.5, 90.0, 93.3, 81.8 | This scoring model may be useful for predicting 2-week outcomes and determining the suitable timing for liver transplantation in patients with non-acetaminophen-related FHF. |
| Biochemical prognostic markers of outcome in non-paracetamol-induced fulminant hepatic failure  Dabos at al.  Transplantation 2004; No. 2, January 2, Vol. 77, 200–205, | Royal Infirmary of  Edinburgh, Edinburgh, Scotland, United Kingdom.  Scottish Liver Transplant Unit  prospective | 59 patients  **age at inclusion**:  41.7±9.3 (underwent LT), 44.1±7.0 (died) 43.7±9.3 (survivors)  **sex:**  M22/F37  **inclusion criteria:**  history of acute liver injury with a cause other than paracetamol overdose and fulfillment of at least two of the five KCH criteria of poor prognosis. Patients were also admitted if they were HE, that is, had developed FHF | **outcome**:  mortality  **mortality**:  15 died.  19 underwent LT  **time of measurement**:  admission  **purpose**:  identify early biochemical markers of clinical outcome in patients with non-paracetamol–induced FHF | **statistical analysis:**  stepwise forward logistic regression  **model:**  1) 0.5×(albumin [g/L])-2×(lactate [mmol/L]) -36×(valine [mmol/L]) -38×(pyruvate[mmol/L])  2) KCC  **validation**: No | The result of the equation < 2 was able to predict death or LT  Coefficient (95% CI):  Albumin -3.4 (-6.1– -0.9)  Lactate 1.3 (0.7–1.9)  Pyruvate -1.1 (-2.0– -0.1)  Valine 0.25 (0.08–0.38)  PT 0.06 (0.1-0.02)  Bilirubin 0.03 (0.06-0.002)  Sensitivity (%), Specificity (%), PPV (%), NPV (%):  1) 94, 86, 91, 86  2) KCC (on admission):  45, 88, 83, 43  KCC (overall):  81, 79, 84, 77 | Identified biochemical markers (used in the model) may be useful in predicting outcome in patients with non-paracetamol–induced FHF and should be evaluated further in a different patient population.  Biochemical criteria on admission had better positive and negative predictive values, sensitivity, and specificity than the KCH criteria on admission and overall. |
| MELD Score as a Predictor of Pretransplant and Posttransplant Survival  in OPTN/UNOS Status 1 Patients  Kremers et al.  HEPATOLOGY 2004; 39:764 –769 | Organ Procurement and Transplantation Network/ United Network for Organ Sharing (OPTN/UNOS)  1 November 1999 - 14 March 2002  prospective | 388 patients  **age at inclusion:**  POD subgroup:  mean 34.4±12.1, median 32 (20-69)  nPOD subgroup:  mean 40.3±13.1, median 39 (19-73)  **sex:**  POD subgroup:  15 (19.7%) M  nPOD subgroup:  103 (33.0%) M  **inclusion criteria:**  onset of stage II HE within 8 weeks of the first symptoms of liver disease, and asterixis, hyperbilirubinemia,  and marked prolongation of the PT (INR) or hypoglycemia | **outcome:**  30-day survival  **mortality:**  66 died,  211 underwent LT  **time of prediction:**  NR  **purpose:**  - to evaluate the ability of the MELD score at listing to predict pretransplant and posttransplant survival for patients listed as UNOS Status 1  - to assess whether the different diagnostic groups of patients listed as Status 1 differ with respect to pretransplant and posttransplant survival. | **statistical analysis:**  Cox model  **model:**  MELD = 3.78×log_e_(bilirubin [mg/dL]  + 11.20×log_e_ (INR) + 9.57×  log_e_(creatinine [mg/dL]) + 6.4  **validation: -** | For an FHF-NA patient with a MELD score of 35.6, the estimated 30-day survival probability was 91% if the patient underwent LT immediately upon being listed. The estimated 30-day survival probability was 58% for a patient awaiting OLT. | Liver allocation within the Status 1 designation may need to be further stratified by diagnosis, and MELD score may be useful for prioritizing FHF-NA candidates. |
| Aetiology and prognostic factors in acute liver failue in India.  Khuroo et al.  Journal of Viral Hepatitis 2003; 10, 224–231 | Liver Unit of Gastroenterology Department at Sher-I-Kashmir Institute of Medical Sciences  India  April 1989–April 1996  retro/prospective NR | 180 patients  **age at inclusion:**  31.1 ± 14.7 (4–65)  **sex:**  69M/111F  **inclusion criteria:**  ALF patients (HE developed  within 8 weeks of the onset of symptoms of liver disease, in a patient with no prior known liver disease) | **outcome:**  mortality  **mortality:**  131 died  **time of measurement:**  admission  **purpose:** prognostic factors of poor outcome in acute liver failure for hepatitis E virus | **statistical analysis:**  stepwise logistic regression  **model:**  non-E aetiology,  prothrombin time >30 s, grade of coma >2, age>40 years  **validation:** No | OR (95%CI):  etiolgy (non-E): 20 (2.3–21.4)  PT (>30): 9.3 (1.3–50.6)  age (>40): 4.7 (1.8–344)  HE (grade>2): 7.5 (3.3–61.9)  Sensitivity (%), Specificity (%), PPV (%), NPV (%), PA (%):  Any 1 factor:  50.9, 76.9, 90.6, 26.3, 55.7  Any 2 factors  84.9, 76.9, 93.8, 52.6, 82.0  Any 3 factors:  90.9, 76.9, 93.0, 83.3, 90.9  All 4 factors:  100, 76.9, 82.4, 100, 88.9 | ALF caused by HEV had a favourable outcome while those caused by NANEH agent had poor outcome. Assessing these variables separately showed significant and contrasting impact of these two variables on survival of patients. Early predictors of a poor outcome were age >40 years, PTT >30 s, grade of coma >2 and non-E aetiology. |
| Prognostic Evaluation of Early Indicators in  Fulminant Hepatic Failure by Multivariate  Analysis  Dhiman et al.  Digestive Diseases and Sciences, 1998; Vol. 43, No. 6: 1311± 1316 | tertiary care center in  northern India  study period: over 5 years  retro/prospective NR | 204 patients population but 186 patients analyzed  **age at inclusion**:  in population: 28.5 (1-75)  **sex:**  98M/106F (population)  91M/95F (analysed)  **inclusion criteria**:  patients with ALF defined according to the criteria of O’Grady et al, ie, onset of HE occurring within 12 weeks of onset of jaundice and further subclassied into hyperacute, acute and subacute liver failure. | **outcome**:  mortality  **mortality**:  126 non-survivors  **time of measurement**:  admission  **purpose**  to study early prognostic  factors based on univariate and multivariate analysis | **statistical analysis:**  logistic regression  **model:**  reression equation, coefficients:  PTT (>100s) 2.41  Raised ICP 2.23  Age (>50 yr) 1.61  Jaundice – HE interval (>7 days) 1.17  HE (gr 3 or 4) 0.77  Bilirubin (≥ 20 mg/dl) 0.24  Constant 1.34  if PTT is > 100 sec, value is 1, otherwise 0;  if raised ICP is present, value is 1, otherwise 0;  if age is > 50 years, value is 1, otherwise 0;  if interval between onset of jaundice and onset of HE >7days, value is 1, otherwise 0;  if grade of HE is 3 or 4, value is 1, otherwise 0;  if total bilirubin leve l is ≥ 20 mg/dl, value is 1, otherwise 0.  final variables:  presence of raised ICP, PTT > 100 sec, age (> 50 yr), onset of HE 7 days after onset of jaundice  **validation**: No |  | Factors adversely affecting the outcome in FHF patients complicating viral hepatitis include presence of overt clinical features of raised ICP at the time of hospitalization, PTT (>100 sec) on admission, age (> 50 years) , and onset of HE seven days after onset of jaundice. |
| Early indicators of prognosis in fulminant hepatic failure: an  assessment of the King’s criteria  Anand et al.  Journal of Hepatology 1997; 26: 62-68 | Liver Unit, Queen Elizabeth Hospital, Birmingham, UK    1990 – 1994  retrospective | 145 patients  **age at inclusion:**  median 31 (18-84)  **sex:**  60M/85F  **inclusion criteria:**  FHF defined as the development of HE within 8 weeks of the onset of symptoms in a patient without previously known liver disease | **outcome:**  mortality  **mortality:**  81 died  **time of measurement:**  admission, peak  **purpose:**  independent indicators of prognosis, risk of death | **statistical analysis:**  stepwise logistic regression  **model:**  1) KCC  2) any of:  Age <20 or >40 years, unfavorable etiology,  jaundice >7 days before HE, PT >50s, PT >l00s,  bilirubin >300 µmol/l  3) log [p/l-p] = (2.248× grade of HE) + (0.0117 × PT in seconds) + (0.0064 × serum creatinine in µmol/l) + (0.045 × white cell count as 10^9^/1) +  (1.4623 only if serum potassium level > 5.5 mmol/l) - 13.241  **validation:** No | PPV (%), NPV (%), PA (%):  1)KCC (at admission):  (POD subgroup): 88, 65, 71  (nPOD subgroup): 79, 50, 68  KCC (peak):  (POD subgroup): 75, 60, 66  (nPOD subgroup): 68, 25, 61  Specificity (%), Sensitivity (%), PA (%):  2) Any 1 indicator: 0 100 61  Any 2 indicators: 0 82 50  Any 3 indicators: 43 82 50  Any 4 indicators: 100 18 50  3) PPV (%), NPV (%):  WBC>20×10^9^: 82%, 74%  Potassium >5.5mmol/l: 85%, 70% | For acetaminophen-  induced FHF patients, KCH criteria remain the mainstay for selecting patients for liver  transplantation. The positive predictive value of these  criteria can possibly be improved by including white  cell count and potassium abnormalities. |
| Fulminant hepatitis in a tropical population Clinical course cause and early predictors of outcome.  Acharya et al.  HEPATOLOGY 1996; 23:1448-1455 | Gastroenterology ward of the All India Institute of Medical Sciences, New Delhi    January 1987 - June 1993  prospective | 423 patients  **age at inclusion**:  14-83  **sex:**  200M/223F  **inclusion criteria**  patients with FHF diagnosed by the presence of HE within 8 weeks of onset of illness | **outcome:**  mortality  **mortality:**  288 (66%) died  **time of measurement:**  admission  **purpose:**  to identify the demographic characteristics, causative spectrum, clinical features, natural course, and predictors of outcome among patients with FHF | **statistical analysis:**  Cox’s proportional hazard regression, and multiple stepwise logistic regression  **model:**  1) logistic reression coefficients:  age ( ≥40yr) 1.3  grade of coma (>2) 0.7  cerebral edema 1.3  infection 1.0  serum bilirubin (≥15 mg/dL) 1.1  PT (≥25s) 1.2    2) *Cox regression coefficients:  cerebral edema 0.87  PT (≥25) 0.43  serum bilirubin (≥15 mg/dL) 0.29  age (≥40 yr) 0.29  **validation:** No | Sensitivity (%), Specificity (%), PPV (%), NPV (%), PA (%):  Any 1 factor*:  56.1, 79.7, 86.3, 44.3, 63.3  Any 2 factors*:  80.9, 79.7, 87.7, 69.9, 80.4  Any 3 factors:*  93, 79.7, 86.0, 89.5, 87.3  All 4 factors*:  92.3, 79.7, 48, 98.1, 81.8 | The prognostic model developed in this study was simple, reliable, rapid, and relevant to patients in developing countries for assessment for LT. |
| Prognostic factor analysis of FHF SFHF in an area endemic for hepatitis  Huo et al.  J of Gastroenterology and Hepatology 1996; 11, 560-565 | Veterans General Hospital Taipei  January 1982 – October 1994  retrospective | 61 patients  **age at inclusion**:  14-83  **sex:**  52M/9F  **inclusion criteria**  ALF complicated with HE developed in patients without pre-existing liver disease or cirrhosis within 2 weeks (FHF) or 2week to 3 months after onset of jaundice (SFHF) | **outcome**:  non-survival  **mortality**:  52 non-survivors  9 survivors  **time of measurement**:  admission, peak values  **purpose**:  To determined the criteria in selecting candidates for OLT | **statistical analysis:** stepwise logistic regression  **model:**  *age>43years,  bilirubin >23mg/dL,  PTT>19s  **validation**: No | Sensitivity (%), Specificity (%), PPV (%), PA (%):  Any 1 indicator  100, 67, 95, 100, 95  Any 2 indicators  69, 100, 100, 36, 74  Any 3 indicators  13, 100, 100, 17, 26 | The indicators may be useful for selecting patients with ALF of various etiologies indicated for OLT and could be a useful tool in the area endemic for HBV infection. A prospective observation is mandatory to justify the validity of these indicators in the future. |
| Early Indicators of Prognosis in Fulminant Hepatic Failure.  O'Grady et al.  Gastroentrology 1989; 97:439-45 | Liver Failure Unit  King’s College School of Medicine and Dentistry,  London,UK  1973-1985  validation: 1986-1987  NR, validation - retrospectively | 588 patients  validation: 175 patients  **age at inclusion**:  NR  **sex:**  NR  **inclusion criteria**  HE developed within 8 wk of the onset of symptoms | **outcome**:  transplantation  **mortality**:  NR  **time of measurement**:  admission, peak  **purpose**:  to identify the factors most likely to indicate a poor prognosis  tested, leading to the construction of models for the selection of patients for LT | **statistical analysis:**  stepwise logistic regression  **model:**  1)  Acetaminophen induced  pH<7.3  or  PT>100s + serum creatinine>300µmol/L +grade III-IV HE:  Non-acetaminophen induced  Age<10 or>40yr, Unfavorable etiology, Jaundice>7days before HE, PTT>50s, Bilirubin>300µmol/L  2)  *Any of:  Age<10 or>40yr, Unfavorable etiology, Jaundice>7days before HE, PTT>50s, Bilirubin>300µmol/L  **validation**: Yes | Sensitivity (%), Specificity (%), PPV (%), PA (%):  1)  Acetaminophen induced  pH<7.3: 49, 99, 95, 81  PT >100s: 79, 67, 72, 71  serum creatinine>300µmol/L:  70, 69, 56, 69  Nonacidotic patients:  PT>100s: 77, 71, 44, 73  serum creatinine>300µmol/L:  77, 58, 35, 63  PT>100s + serum creatinine> 300µmol/L: 55, 87, 55, 80  PT>100s + serum creatinine> 300µmol/L+grade III-IV HE:  45, 94, 67, 83  Non-Acetaminophen related  Age<10 or>40yr: 50, 90, 96 57  Unfavorable etiology: 80, 60, 90, 76  Jaundice>7days before HE:  82, 90, 97, 83  PTT>50s: 75, 90, 97, 78  PTT>100s: 34, 100, 100, 46  Bilirubin>300µmol/L: 84, 70, 93, 81  2) Patients with PTT<100s  Any 2 indicators*  97, 80, 93, 92  Any 3 indicators*  93, 90, 96, 92  Any 4 indicators*  59, 100, 100, 67  Patients not treated by LT:  Any 1 indicator*  100, 20, 80, 81  Any 2 indicators  94, 80, 94, 90  Any 3 indicators  91, 90, 97, 90  Any 4 indicators  59, 100, 100, 69 | Criteria for referring patients with FHF for LT were established and it is anticipated that these will improve the speed and accuracy of the selection of appropriate candidates. |
| Multivariate analysis of prognostic factors in fulminant hepatitis B  Bernuau J. et al.  HEPATOLOGY 1986; Vol. 6, No. 4: 648-651 | Liver Unit at Hopital Beaujon  January 1972 – December 1981  retro/ prospective NR | 115 patients  **age at inclusion**:  mean 40 (15-77)  **sex:**  43M/72F  **inclusion criteria:**  fulminant hepatitis B diagnosed based on: histlogically proven acute hepatitis, sever coagulation disorders, HE within 2 months after the onset of jaundice, presence of IgManti-HBc after onset of HE | **outcome**:  survival 90-days after onset of jaundice  **mortality:**  89 died (77.4%)  **time of measurement**:  at the data of testing for IgM anti-HBc  **purpose**:  assessment of prognostic factors in patients with fulminant hepatitis B | **statistical analysis:**  Cox’s proportional hazard regression  **model:**  factor V level  age  absence of detectable HBsAg  AFP  **validation**: No | - | In patients with fulminant hepatitis B the absence of HBsAg in serum has an independent, favourable prognostic value. |
| Prediction of fatality in Fulminant Hepatic Failure  Christensen E. et al.  Scand J Gastroenterol 1984; 19:90-96 | Intensive Liver Unit of Rigshospitalet, Denmark  Period NR  retro/ prospective NR | 23 patients  **age at inclusion**:  31.5 (20-72) non-survivors  26 (17-49) survivors  **sex:**  9M/14/F  **inclusion criteria**:  patients with FHF | **outcome**:  mortality  **mortality:**  20 died  **time of measurement**: admission  **purpose**:  prediction of non-survival | **statistical analysis:**  discriminative analysis with stepwise addition  **model:**  1) full score; significant discriminant function coefficients:  taurine (24^-14^C) cholic acid conjugation -1.15  sex -0.42  pregnancy -0.85  carbamide 0.024  ALP 0.0015  glycolithocholic acid suplhate -0.040  non-A non-B viral hepatitis -0.74  age 0.012  glycine (24^-14^C) cholic acid conjugation -0.53  duration of history 0.0044  halothane hepatitis -0.27  grade of HE 0.087  leukocyte count 0.013  constant 0.18    2) reduced score; significant discriminant function coefficients:  disulfiram hepatitis 0.83  blood glucose concentration 0.089  duration of history 0.0081  leukocyte count 0.032  age 0.015  prothrombine index -1.28  sex -0.27  viral hepatitis B 0.31 serum potassium 0.12  serum albumin -0.0019  blood type O -0.25  constant -0.45  **validation**: Yes  discriminant score validated by an unbiased method in which each patient is classified on the basis of the other patients’ data | The discriminant power of the estimated discriminant function was studied by allocating each patient on the basis of all the other patients (leaving current patient out method).  based on Bayes theorem for discriminant analysis  95% CI, Sensitivity (%), Specificity (%)  1) 0.41-0.59, 100-95, 100  2) 0.4-0.6, 90-75, 77-92 | It is suggested that the discriminant score is used to select patients with very low probability of survival for LT or liver assistance procedures of unknown value. |

ALF, acute liver failure; ATT, antituberculosis therapy ; AUROC = AUC Area under the receiver operating characteristic (ROC) curve; CI, confidence interval; F, female; FHF, fulminant hepatic failure; HR, hazard ratio;

HE, hepatic encephalopathy; ICP, intracranial pressure; KCC, King’s Collage Criteria; LT, liver transplantation; MELD; Model for End-Stage Liver Disease; NLR, negative likelihood ratio; NPV, negative predictive value; OLT – orthotopic liver transplantation; OR odds ratio; PA, predictive accuracy; PLR, positive likelihood ratio; PPV, positive predictive value; POD – paracetamol overdose group; nPOD – non paracetamol overdose group; PT, prothrombin time; ROC, receiver-operating characteristic
