# Supplementary material for: Prediction of Poor Outcome in Patients with Acute Liver Failure—Systematic Review of Prediction Models
Source: PLoS One. 2012 Dec 14;7(12):e50952. doi: 10.1371/journal.pone.0050952 (PMC3522683; doi:10.1371/journal.pone.0050952)
Supplement: Table S2 — Summary of input variables used in the studies. (DOC) [file pone.0050952.s003.doc]

**Supplement Table S2: Summary of input variables used in the studies (U/M = included in uni/multi variate analysis; - not significant, + significant; F+/- included in the final model / not included in the final model)**

|  | Bretherick et al  2011 | Kumar  et al.  2010 | Yamagishi et al.  2009 | Hadem et al.  2008 | Dhiman et al. 2007 | Pelaez-Luna  et al. 2006 | Schmidt et al. 2006 | Taylor et al. 2006 | Dabos et al. 2005 | Miyake et al. 2005 | Dabos at al. 2004 | Khuroo et al. 2003 | Dhiman et al. 1998 | Anand et al. 1997  POD | Anand et al. 1998 nPOD | Acharya et al. 1996 | Huo  et al. 1996 | O'Grady et al. 1989 POD | O'Grady et al. 1989 nPOD | Bernuau et al. 1986 | Christensen  et al. 1984 |
| --- | --- | --- | --- | --- | --- | --- | --- | --- | --- | --- | --- | --- | --- | --- | --- | --- | --- | --- | --- | --- | --- |
| Acetate |  |  |  |  |  |  |  |  | U+ M+ F- |  |  |  |  |  |  |  |  |  |  |  |  |
| AFP |  |  |  |  |  |  |  |  |  |  |  |  |  |  |  |  | U+ M- F- cc>400ng/ml |  |  | U+M+F+ |  |
| Age |  | U- F- | U- M- F- cc<11or>40 | U- F- | U+ M+ F+ cc>=50 | U- F- |  | U- F- |  | M- F- c>45 |  | U+ M+ F+ cc>40 | U+ M+ F+ c>50 | U- M- F- | U- M- F- | U+ M+ F+ cc<40 | U+ M+ F+ c>43 | M+ F- | M+ F+ c<11 >40 | U+M+F+ | U- M+ F+ |
| Alanine |  |  |  |  |  |  |  |  | U+ M+ F- |  | U+ M- F- |  |  |  |  |  |  |  |  |  | U- F- |
| Albumin |  | U- F- |  |  | U- F- | U+ M- F- |  |  | U+ F- | M- F- c>3g/dl | U+ M+ F+ |  | U- F- c<3.0g/dl |  |  |  | U- F- |  |  |  | U- M+ F+# |
| ALP | U+^M-^  F-^ U-# | U- F- |  | U+ F- | U- F- |  |  | U+ F- |  |  |  | U- F- |  |  |  |  |  | U- M- F- | U- M- F- |  | U- M+ F+^ |
| ALT | U-^  U+#M+#  F+# | U- F- | U+ M- F- | U+ F- | U- F- |  |  | U+ M+ F+ cc<2600 IU/L |  |  |  | U- F- | U- F- c>10x normal |  |  |  | U- F- |  |  | U-M-F- | U- F- |
| Ammonia |  |  | U- M- F- | U+ F- |  |  |  |  |  |  |  |  |  |  |  |  |  |  |  |  |  |
| Amylase |  |  |  |  |  |  |  |  |  |  |  | U- F- |  |  |  |  |  |  |  |  |  |
| Anti-HBs |  |  |  |  |  |  |  |  |  |  |  |  |  |  |  |  |  |  |  | U-M-F- |  |
| APACHE II |  |  |  |  |  | U+ M+ F+ |  |  |  |  |  |  |  |  |  |  |  |  |  |  |  |
| Ascites |  |  |  |  | U- F- |  |  |  |  |  |  |  | U- F- |  |  |  |  |  |  |  |  |
| AST |  | U- F- |  | U+ F- | U- F- |  |  | U- F- |  |  |  |  |  |  |  |  |  | U- M- F- | U+* M- F- |  |  |
| AST:ALT ratio |  |  |  |  |  |  |  |  |  |  |  |  |  |  |  |  | U- F- |  |  |  |  |
| Bicarbonate |  |  |  |  |  | U+ M- F- |  |  | U- F- |  |  |  |  |  |  |  |  |  |  |  |  |
| Bilirubin total | U+M+F+ | U+ M+ F+ c>= 10,8mg/dL | U+ M- F+ cc>17mg/dl | U+ M+ F+ cc >140µmol/L | U+ M- F- cc >=20mg/dl | U+ M+ F+ |  | U- F- |  | M- F- c>15mg/dl | U+ M+ F- | U- F- | U+ M- F- c >=20mg/dl | U- M- F- | U- M- F- | U+ M+ F+ c>=15 mg/dl | U+ M+ F+ c>23mg/dl | U+ M+** F- | U+ M+ F+ cc>300µmol/L |  | U+ F- |
| Bilirubin T/D ratio |  |  |  |  |  |  |  |  |  | U+ M+ F+ c>2 |  |  |  |  |  |  |  |  |  |  |  |
| Blood gas (PaO2, pcP2, O2) |  |  |  |  |  |  |  |  |  |  |  | U- F- |  |  |  |  |  |  |  |  |  |
| Blood type O |  |  |  |  |  |  |  |  |  |  |  |  |  |  |  |  |  |  |  |  | U- M+ F+# |
| Body weight |  |  |  |  |  |  |  |  |  |  |  |  |  |  |  |  |  |  |  |  | U- F- |
| Calcium |  |  |  |  |  |  |  |  | U+ M+ F- |  |  |  |  |  |  |  |  |  |  |  |  |
| Carbamide |  |  |  |  |  |  |  |  |  |  |  |  |  |  |  |  |  |  |  |  | U- M+ F+^ |
| Cerebral edema |  | U+ M- F- |  |  | U+ M+ F+ |  |  |  |  |  |  | U+ M- F- |  |  |  | U+ M+ F+ |  |  |  |  |  |
| CHDF continuous hemodiafiltration |  |  | U- F- |  |  |  |  |  |  |  |  |  |  |  |  |  |  |  |  |  |  |
| Citrulline |  |  |  |  |  |  |  |  |  |  | U- M- F- |  |  |  |  |  |  |  |  |  |  |
| Cholesterol |  |  |  |  |  |  |  |  |  |  |  |  |  |  |  |  | U+ M- F- |  |  |  |  |
| Cholic acid coniugation total |  |  |  |  |  |  |  |  |  |  |  |  |  |  |  |  |  |  |  |  | U+ F- |
| Cholic acid coniugation glycine |  |  |  |  |  |  |  |  |  |  |  |  |  |  |  |  |  |  |  |  | U+ M+ F+ ^ |
| Cholic acid coniugation taurine |  |  |  |  |  |  |  |  |  |  |  |  |  |  |  |  |  |  |  |  | U- M+ F+^ |
| Cholic acid coniugation sulphate |  |  |  |  |  |  |  |  |  |  |  |  |  |  |  |  |  |  |  |  | U- F- |
| Corticosteroids administration |  |  |  |  |  |  |  |  |  |  |  |  |  |  |  |  |  |  |  | U-M-F- |  |
| Creatinine | U+M+F+ | U- F- |  | U- F- | U+ M+ F+ cc≥1.5 mg/dL | U+ M- F- |  | U+cont, U-cat M+ F+ cc>2.0mg/dl | U- F- | M- F- c>2mg/dl |  |  | U- F- c>=3mg/dl | U-^U+#  M+* F+ | U- M- F- |  | U+ M- F- | U+ M+ F+ cc>300µmol/L | U+** M- F- |  | U- F- |
| CTLV/SLV |  |  | U+ M+ F+ cc<0,8 |  |  |  |  |  |  |  |  |  |  |  |  |  |  |  |  |  |  |
| D-dimers |  |  |  |  |  |  |  |  |  |  | U- M- F- |  |  |  |  |  |  |  |  |  |  |
| Duration of jaundice |  |  |  |  | U+ M- F- cc>5,5days |  |  |  |  |  |  |  |  |  |  |  |  |  |  |  |  |
| Duration of history |  |  |  |  |  |  |  |  |  |  |  |  |  |  |  |  |  |  |  |  | U- M+ F+ |
| Etiology |  |  | U- M- F- |  |  |  |  |  |  | U+ M+ F+ |  | U+ M+ F+ |  |  | U- M- F- |  |  |  | M+ F+ |  | U- M+ F+ |
| Factor V |  |  |  | U- F- |  |  |  |  |  |  |  |  |  |  |  |  |  |  |  | U+M+F+ |  |
| Fatty liver of pregnency |  |  |  |  |  |  |  |  |  |  |  |  |  |  |  |  |  |  |  |  | U- F- |
| Fibrinogen |  |  |  |  |  |  |  |  |  |  |  |  |  |  |  |  |  |  |  | U-F- |  |
| Fresh frozen plasma infusion |  |  |  |  | U- F- |  |  |  |  |  |  |  | U- F- |  |  |  |  |  |  |  |  |
| Galactose elimination capacity |  |  |  |  |  |  |  |  |  |  |  |  |  |  |  |  |  |  |  |  | U- F- |
| Gastroeintestinal bleeding |  |  |  |  |  |  |  |  |  |  |  | U- F- |  | U+** M-** F- | U- M- F- |  |  |  |  |  |  |
| GLASGOW score |  |  |  |  |  | U+ M- F- |  |  |  |  |  |  |  |  |  |  |  |  |  |  |  |
| Glucose |  |  |  |  |  |  |  |  |  |  |  |  |  |  |  |  |  |  |  |  | U- M+ F+# |
| Glycocholic acid |  |  |  |  |  |  |  |  |  |  |  |  |  |  |  |  |  |  |  |  | U- F- |
| Glycolithocholic acid sulphate |  |  |  |  |  |  |  |  |  |  |  |  |  |  |  |  |  |  |  |  | U- M+ F+^ |
| Glycine |  |  |  |  |  |  |  |  |  |  | U+* U-** M- F- |  |  |  |  |  |  |  |  |  |  |
| H+ |  |  |  |  |  |  |  |  | U- F- |  |  |  |  |  |  |  |  |  |  |  |  |
| HBsAg |  |  |  |  | U- F- |  |  |  |  |  |  |  | U- F- |  |  |  |  |  |  | U+M+F+ |  |
| HE | M+F+ | U+ M+ F+ cIII-IV | U- M- F- | U+ F- | U- M+ F+, O c III/IV |  |  | U- F- cIII-IV |  | U+ M+ F+ cII vs II-IV |  | U+ M+ F+ cc 3,4 | U+ M- F- c III/IV | U+** M+** F+** | U+** M-** F- | U+ M+* F+* ccIII-IV | U- F- cIII-IV | M+* F+ccIII-IV | M+* F- | U-M-F- | U- M+ F+^ |
| Heart rate |  |  |  |  |  | U+ M- F- |  |  |  |  |  |  |  |  |  |  |  |  |  |  |  |
| Hematocrit |  |  |  |  |  | U+ M- F- |  |  |  |  |  |  |  |  |  |  |  |  |  |  |  |
| Hemodialysis |  |  |  |  |  |  |  | U- F- |  |  |  |  |  |  |  |  |  |  |  |  |  |
| Hemoglobin | U+M-^ F-^  M+#F+# |  |  | U+ F- | U- F- | U+ M- F- |  | U- F- | U+ M+ F- | M- F- c>10,0g/dl |  | U- F- |  |  |  |  |  |  |  |  |  |
| Histidine |  |  |  |  |  |  |  |  |  |  | U-*, U+**, M- F- |  |  |  |  |  |  |  |  |  |  |
| ICP raised |  |  |  |  |  |  |  |  |  |  |  |  | U+ M+ F+ |  |  |  |  |  |  |  |  |
| Infection |  |  |  |  |  |  |  |  |  |  |  |  |  | U+** M-** F- | U+, M-, F- | U+ M+* F+* |  |  |  |  |  |
| INR |  |  | U- cc>3,5, M- F- | U+ M-F- |  | U+ M- F- |  | U- F- | U+ F- |  |  |  |  |  |  |  |  |  |  |  | U- M+ F+# |
| Interval ATT - ALF |  | U- F- |  |  |  |  |  |  |  |  |  |  |  |  |  |  |  |  |  |  |  |
| Interval hospital admission to study enrollment |  |  |  |  |  |  |  | U- F- |  |  |  |  |  |  |  |  |  |  |  |  |  |
| Interval onset of symptoms to the diagnosis |  |  |  |  |  |  |  |  |  | M- F- c>7 |  |  |  |  |  |  |  |  |  |  |  |
| Interval onset of symptoms to icterus |  |  |  |  |  |  |  |  |  |  |  |  |  |  |  | U- F- |  |  |  |  |  |
| Interval onset of symptoms to HE |  |  |  |  |  |  |  |  |  |  |  |  |  |  |  | U- F- |  |  |  |  |  |
| Interval onset of symptoms to study enrolment |  |  |  |  |  |  |  | U- F- cc>21dsys |  |  |  |  |  |  |  |  |  |  |  |  |  |
| Interval jaundice-HE |  | U- F- | U+, M-, F- c>  1week |  | U+ M+ F+ cc>  7days |  |  |  |  |  |  | U+ M- F- cc<=  7days | U+ M+ F+ c>  7 days |  | U- M- F- | U- F- | U- F- c>28days |  | M+ F+ | U-M-F- |  |
| Intubated |  |  |  |  |  |  |  | U+ M+ F+ |  |  |  |  |  |  |  |  |  |  |  |  |  |
| KCC |  | U- F- | U+ M- F- | U+ F- |  |  | M+ F+ |  |  |  |  |  |  |  |  |  |  |  |  |  |  |
| Lactate |  |  |  | U+ M+ F+ cc3,5mmol/L |  |  | M+ F+ c>4mmol/L |  | U+ M+ F- |  | U+ M+ F+ | U- F- |  |  |  |  |  |  |  |  |  |
| Lamivudine and/or IFN interferon |  |  | U- F- |  |  |  |  |  |  |  |  |  |  |  |  |  |  |  |  |  |  |
| Leukocytes |  |  |  |  |  | U+ M- F- |  |  |  |  |  |  |  |  |  |  |  |  |  |  | U- M+ F+ |
| Liver size (in percussion space) |  |  |  |  |  |  |  |  |  |  |  |  |  |  |  | U+ M- F- c<2 |  |  |  |  |  |
| Leucine |  |  |  |  |  |  |  |  |  |  | U-* U+** M- F- |  |  |  |  |  |  |  |  |  |  |
| MAP |  |  |  |  |  | U+ M- F- |  |  |  |  |  |  |  |  |  |  |  |  |  |  |  |
| MELD |  | U+ con; for cat>33 NR | U- cc>=30 M- F- | U+ F- cc>32 | U+ F- cc≥33 |  |  | U- F-cc≥35 |  |  |  |  |  |  |  |  |  |  |  |  |  |
| N-acetyl cysteine treatment |  |  |  |  |  |  |  |  |  |  |  |  |  | U- M- F- |  |  |  |  |  |  |  |
| PE plasma exchange |  |  | U- F- |  |  |  |  |  |  |  |  |  |  |  |  |  |  |  |  |  |  |
| pH |  |  |  |  |  |  |  | U- F- |  |  |  | U- F- |  | U+ M+** F+ | U- M- F- |  |  | U-* M+* F+ cc<7.30 | U-* M+* F- |  |  |
| Phenylalanine |  |  |  |  |  |  |  |  | U+ M+ F+ |  |  |  |  |  |  |  |  |  |  |  |  |
| Phosphate |  |  |  |  |  |  |  | U- F- | U- F- |  |  |  |  |  |  |  |  |  |  |  |  |
| Plasma phenazone clearance |  |  |  |  |  |  |  |  |  |  |  |  |  |  |  |  |  |  |  |  | U- F- |
| Platelet count | U-^  U+#M+#F+# |  |  |  |  |  |  | U- F- |  | M- F- c>10x103/mm3 |  | U- F- |  | U+^** M- F- | U- M- F- |  |  | U+** M- F- | U- M- F- |  |  |
| Potassium | U+M-^  F-^M+#  F+# |  |  |  |  |  |  |  |  |  |  | U- F- |  | U-^U+# M+** F+cc>5.5mmol/L | U- M- F- |  |  |  |  |  | U-, M+ F+# |
| Pressors |  |  |  |  |  |  |  | U+ M+ F+ |  |  |  |  |  |  |  |  |  |  |  |  |  |
| Pregnancy |  | U- F- |  |  |  |  |  |  |  |  |  | U- F- |  |  |  |  |  |  |  |  | M+ F+^ |
| PT | U+M+F+ | U+ M+ F+ c>=26s | U- F- | U- F- | U+ M+ F+ cc >=35s | U+ M- F- |  |  |  | M- F- c>=10% | U+*, U-**, M+ F- | U+ M+ F+ cc>30s | U+ M+ F+ c>100s | U+ M+** F+ | U+** M+** F+  c>75s | U+ M+ F+ c>=25s | U+ M+ F+ c>19s | U+ M+* F+ cc>100s | U+ M+ F+ cc>100s, 50s |  |  |
| Pyruvate |  |  |  |  |  |  |  |  | U+ M+ F+ |  | U+ M+ F+ |  |  |  |  |  |  |  |  |  |  |
| Race/ethnicity |  |  |  |  |  |  |  | U- F- |  |  |  |  |  |  |  |  |  |  |  |  |  |
| Renal failure |  |  |  |  |  |  |  |  |  |  |  | U+ M- F- |  |  |  |  |  |  |  |  |  |
| Respiratory rate |  |  |  |  |  | U+ M- F- |  |  |  |  |  |  |  |  |  |  |  |  |  |  |  |
| SAPS-III |  |  |  | U+ F- |  |  |  |  |  |  |  |  |  |  |  |  |  |  |  |  |  |
| Sepsis |  |  |  |  |  |  |  |  |  |  |  | U+ M- F- |  |  |  |  |  |  |  |  |  |
| Sex |  | U- F- | U- F- | U- F- | U- F- |  |  | U+ F- |  | M- F- |  | U+ M- F- | U- F- | U- M- F- | U- M- F- | U- F- | U- F- |  |  | U-M-F- | U- M+ F+ |
| SIRS |  |  |  |  |  |  |  |  |  | U+ M+ F+ |  |  |  |  |  |  |  |  |  |  |  |
| Sites of tuberculosis § |  | U- F- |  |  |  |  |  |  |  |  |  |  |  |  |  |  |  |  |  |  |  |
| SLV |  |  | U- F- |  |  |  |  |  |  |  |  |  |  |  |  |  |  |  |  |  |  |
| Sodium | U- |  |  | U- F- | U- F- |  |  |  | U+ F- |  |  | U- F- |  | U- M- F- | U- M- F- |  |  | U- M- F- | U- M- F- |  |  |
| Steroid therapy |  |  | U- F- |  |  |  |  |  |  |  |  |  |  |  |  |  |  |  |  |  |  |
| Time prior to HE |  |  | M- F- |  |  |  |  |  |  |  |  |  |  |  |  |  |  |  |  |  |  |
| Treatment (costicosteroids, extracorporeal circulation, exchane transfusions, mannitol, soludactone, heparin) |  |  |  |  |  |  |  |  |  |  |  |  |  |  |  |  |  |  |  | U-M-F- |  |
| Urea | U-^  U+#M+#F+# | U- F- |  |  |  |  |  |  |  |  |  |  |  | U- M- F- | U- M- F- |  |  | U- M- F- | U- M+** F- |  |  |
| Valine |  |  |  |  |  |  |  |  |  |  | U+ M+ F+ |  |  |  |  |  |  |  |  |  |  |
| WBC | U+M-^  M+#F+ |  |  | U+ F- | U- F- |  |  | U- F- |  |  |  | U- F- | U- F- c<4000/mm3 or >18000/mm3 | U+^** M+** F+ | U- M- F- |  | U- F- | U+* M- F- | U+* M- F- |  |  |

c: categorical variable; cc = variable considered as both continuous and categorical

§ Sites of tuberculosis: lymph node, pleuropulmonary, abdominal, disseminated, bone, meningeal, pericardial, empirical

Bretherick et al.: reported significances for both a peripheral hospital and Liver Transplantation Centre; except ^only 1^st^ model, #only 2^nd^ model

Dabos et al 2004: (separate analysis for surv vs death* and surv vs death+LT**) reported separately only when the results for both subgroups differs

Miyake et al.: 4 models proposed, variable for model on day1

Acharya et al.: reported for both log regression model and cox; except * when significant only in log reg model

Huo et al.: reported for both models

O’Grady et al.: reported significances for both admission an peak values; except *only for admission value, ** only for peak value

Christiensen et al.: reported significances for both models; except ^only 1st model, #only 2nd model

Anand et al.: reported significances for both a peripheral hospital and Liver Transplantation Centre; except ^only for peripheral hospital, #only for Liver Transplantation Centre; reported both admission an peak values; except * for admission value, ** for peak value
