# Supplementary material for: Prediction of Poor Outcome in Patients with Acute Liver Failure—Systematic Review of Prediction Models
Source: PLoS One. 2012 Dec 14;7(12):e50952. doi: 10.1371/journal.pone.0050952 (PMC3522683; doi:10.1371/journal.pone.0050952)
Supplement: Table S3 — Quality assessment scores of the included studies. (DOC) [file pone.0050952.s004.doc]

| study | methodological score | 1A. events per variable sufficient in the final model | 1B. n reported | 1C. n-events reported | n-variables reported | 2. included population representative of the population described by the aim /conclusion | 3. model validation | I.1. setting described | I.2. pros/retrospective reported | I.3. patients characteristics described | II.1.1. variables defined | II.1.2. reason for initial variables reported | II.1.3. important variable included | II.1.4. continuous-categorical | II.2.1. missing data reported | II.2.2. handling missing data | II.3. outcome defined | III.1. intended use of model reported | III.2.1. type described | III.2.2. derivation/calibration reported | III.2.3. can use model | III.3. ≥2 performance measures reported | III.3.1. CI / SE reported | III.4. probabilities for ≥2 specific groups of patients reported | III.5. compared to other model | III.6. contribution of the most important item reported | reporting score |
| --- | --- | --- | --- | --- | --- | --- | --- | --- | --- | --- | --- | --- | --- | --- | --- | --- | --- | --- | --- | --- | --- | --- | --- | --- | --- | --- | --- |
| Bretherick et al. 2011 | 10 | Y | Y | Y | Y | Y | N | Y | Y | Y | Y | N | Y | Y | Y | Y | Y | Y | Y | Y | N | N | Y | N | N | Y | 28 |
| Kumar et al. 2010 | 9 | P | Y | Y | Y | Y | N | Y | Y | Y | P | N | Y | P | N | N | Y | Y | Y | Y | Y | Y | P | Y | P | Y | 28 |
| Yamagishi et al. 2009 | 9 | P | Y | Y | Y | Y | N | Y | Y | Y | Y | Y | Y | P | N | N | Y | Y | Y | Y | Y | Y | P | N | P | P | 28 |
| Hadem et al. 2008 | 10 | Y | Y | Y | Y | Y | N | Y | Y | Y | Y | N | Y | P | N | N | Y | Y | N | N | Y | Y | Y | N | Y | Y | 25 |
| Dhiman et al. 2007 | 9 | P | Y | Y | Y | Y | N | Y | Y | Y | Y | P | Y | P | N | N | Y | Y | Y | Y | Y | Y | Y | N | Y | Y | 30 |
| Pelaez-Luna et al. 2006 | 10 | Y | Y | Y | Y | Y | N | Y | Y | Y | P | N | Y | Y | N | N | Y | Y | Y | Y | Y | N | P | N | Y | Y | 26 |
| Schmidt and Larsen 2006 | 10 | Y | Y | Y | Y | Y | N | Y | N | Y | Y | Y | Y | Y | N | N | Y | Y | Y | Y | Y | N | N | N | P | P | 24 |
| Taylor et al. 2006 | 7 | N | Y | Y | Y | P | N | Y | Y | Y | Y | P | Y | P | N | N | Y | Y | Y | N | Y | Y | N | N | Y | Y | 26 |
| Dabos et al. 2005 | 11 | P | Y | Y | Y | Y | Y | Y | N | N | Y | N | P | Y | Y | Y | Y | Y | Y | Y | Y | N | N | N | P | Y | 24 |
| Miyake et al. 2005 | 12 | Y | Y | Y | Y | Y | Y | Y | Y | Y | Y | N | Y | N | N | N | Y | Y | Y | Y | Y | N | N | N | N | Y | 22 |
| Dabos et al. 2004 | 9 | P | Y | Y | Y | Y | N | P | Y | N | Y | N | P | Y | Y | Y | Y | Y | Y | Y | Y | N | N | N | P | Y | 25 |
| Kremers et al. 2004 | 10 | Y | Y | Y | Y | Y | NA | Y | N | P | Y | NA | Y | Y | N | N | Y | Y | Y | NA | Y | N | NA | NA | N | NA | 17 |
| Khuroo et al. 2003 | 10 | Y | Y | Y | Y | Y | N | Y | N | Y | Y | N | Y | N | N | N | Y | Y | Y | Y | Y | N | Y | N | N | Y | 22 |
| Dhiman et al. 1998 | 10 | Y | Y | Y | Y | Y | N | P | N | Y | Y | N | Y | N | N | N | Y | Y | Y | P | Y | N | N | N | N | Y | 18 |
| Anand et al. 1997 | 10 | Y | Y | Y | Y | Y | N | Y | Y | Y | N | Y | Y | P | Y | Y | Y | Y | Y | Y | Y | N | N | NA | P | Y | 28 |
| Acharya et al. 1996 | 10 | Y | Y | Y | Y | Y | N | Y | Y | Y | Y | N | Y | P | N | N | Y | Y | Y | P | P | N | N | N | N | Y | 21 |
| Huo et al. 1996 | 8 | N | Y | Y | Y | Y | N | Y | Y | Y | Y | N | Y | P | N | N | Y | Y | Y | P | Y | N | N | N | N | Y | 22 |
| O'Grady et al. 1989 | 11 | P | Y | Y | Y | Y | Y | Y | N | N | Y | N | Y | N | Y | Y | Y | Y | Y | Y | Y | N | N | N | NA | N | 20 |
| Bernuau et al. 1986 | 10 | Y | Y | Y | Y | Y | N | Y | N | N | Y | Y | Y | Y | Y | N | Y | Y | Y | Y | Y | N | N | N | NA | N | 22 |
| Christensen et al. 1984 | 10 | N | Y | Y | Y | Y | Y | P | N | Y | Y | N | Y | P | N | N | Y | Y | Y | Y | Y | N | N | N | NA | P | 19 |

**Supplement Table S3**: Quality assessment scores of the included studies

II.1.4. If no reason is given for converting continuous to categorical but AUC is reported = P, if clinical explanation is given = Y

III.2.1. Reporting of decision rule / tree, even without calling it by name, is sufficient = Y

III.3.1. If reported e.g. AUC and OR and CI/SE reported for only one of them = P, for both = Y, not reported =N

III.3. Performance measures taken from at least 2 of the groups: 1) sens/spec/PPV/NPV 2) AUC 3) calibration Hosmer-Lemeshow, Brier score 4) accuracy Brier score, R^2^

III.4 If probabilities explicitly reported or number of death expressed in % + sens/spec/PPV/NPV reported = Y

III.5. If AUC to compare the models reported = Y; if only sens/spec/PPV/NPV reported = P

III.6. If paper reports % sens/spec/PPV/NPV = Y; beta of regression analysis = Y, if only AUC = N
